# Undiagnosed Primary Sjögren's Syndrome With Pleural Involvement

**DOI:** 10.7759/cureus.45981

**Published:** 2023-09-26

**Authors:** Rui L Fernandes, Ana C Henriques, Angela Ghiletchi, Maria I Correia, Teresa Faria

**Affiliations:** 1 Internal Medicine, Hospital Dr. Nélio Mendonça, Funchal, PRT; 2 Internal Medicine, Centro Hospitalar Lisboa Central, Lisbon, PRT

**Keywords:** sjögren’s syndrome, rheumatology, lung involvement, pleural effusion, general internal medicine

## Abstract

Sjögren's syndrome (SS) is a systemic autoimmune disorder characterized by the lymphocytic infiltration of the exocrine glands, primarily the salivary and lacrimal glands, resulting in the dryness of the eyes and mouth. However, Sjögren's syndrome is not limited to glandular involvement, as it can also affect various other organ systems, leading to a wide range of extraglandular manifestations and delaying the diagnosis. In this scenario, a high level of suspicion is required.

## Introduction

Sjögren's syndrome (SS) is a chronic autoimmune inflammatory disease that is often not diagnosed promptly. It primarily affects the exocrine glands, leading to the destruction of salivary and lacrimal cells and causing classic symptoms such as dry mouth (xerostomia) and dry eyes (xerophthalmia) [1]. In addition to these classical symptoms, SS has multiple systemic manifestations including fatigue, polyarthritis, vasculitis, renal involvement, lymphoma, and lung involvement [2]. The pulmonary manifestations of SS are diverse and encompass conditions such as airway abnormalities and interstitial lung disease (ILD). However, these lung-related aspects are not extensively studied, despite affecting approximately one-fifth of primary Sjögren's syndrome (pSS) patients. This lack of research results in significant clinical implications that require further investigation [3]. We present a case of a 65-year-old female with a previous diagnosis of fibromyalgia, who was hospitalized for pneumonia complicated with pleural effusions. After an extensive investigation, a diagnosis of primary Sjögren's syndrome (pSS) with pleural involvement was made. This highlights the expanding understanding of Sjögren's syndrome and its potential extraglandular manifestations. This case report was previously presented as a meeting abstract at the 27th Portuguese Congress of Internal Medicine on 2-5 October 2021.

## Case presentation

**Figure 1:**
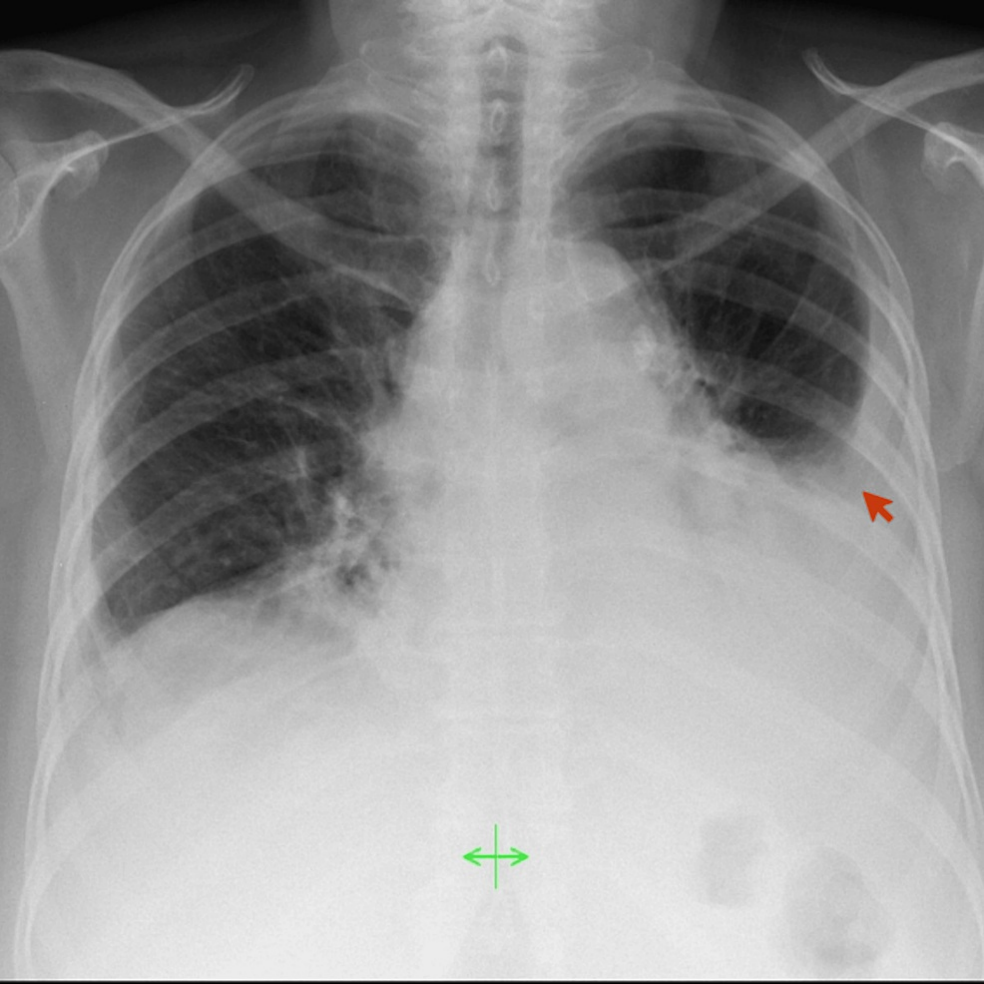
Chest X-ray showing bilateral pleural effusion but more evident on the left side, as indicated by the red arrow

We present a case of a 65-year-old female with a previous diagnosis of fibromyalgia, who was admitted to the emergency department for dyspnea, cough, and fever. Recently, she was hospitalized two months ago due to community-acquired pneumonia for which she completed seven days of amoxicillin/clavulanic acid empirically. Physical examination revealed a peripheral oxygen saturation (SpO_2_) of 90%, a normotensive blood pressure, and a body temperature of 38.6°C. Additionally, she reported progressively worsening xerostomia, xerophthalmia, asthenia, adynamia, and symmetrical non-deformative arthritis involving the small joints of both hands. Initial laboratory test results (Table 1, at admission) revealed normochromic normocytic anemia, leukocytosis with left shift, thrombocytosis, hypokalemia, and an elevated C-reactive protein (CRP). Chest X-ray showed bilateral pleural effusion, more prominent on the left side (Figure 1).

**Table 1:** Laboratory results of the case WBC, white blood cell; Cr, creatinine; K, potassium; F, phosphorus; Ca, calcium; CRP, C-reactive protein; ESR, erythrocyte sedimentation rate; ANA, antineutrophil antibodies; SSA, Sjögren's syndrome-related antigen A; SSB, Sjögren's syndrome-related antigen B; dsDNA, double-stranded DNA; RF, rheumatoid factor

	Admission	Evolution	Discharge	Normal range
WBC (/μL)	13.600	8.400	7.100	4.200-10.800
Neutrophils (%)	81.4	90.6	78.9	-
Hemoglobin (g/dL)	10.5	7.5	8.6	13.7-17.3
Platelets (/μL)	542.000	372.000	441.000	144.000-440.000
Cr (mg/dL)	0.7	0.69	0.7	0.70-1.20
K+ (mEq/L)	3.1	2.8	4	3.50-5.10
F+ (mEq/L)	-	<1	3.2	2.40-4.70
Ca+ (mg/dL)	-	6.34	9.3	8.90-10.30
Vitamin D (ng/mL)	-	12	-	<20
CRP (mg/L)	235.72	172.57	45	<6.10
ESR (mm/hour)	-	83	-	<30
Haptoglobin (mg/dL)	-	407.00	-	30.00-200.00
Ferritin (ng/mL)	-	2563	-	13.0-150.0
RF (IU/mL)	-	9.7	-	<16
ANA result	-	Positive	-	
ANA title	-	1/640	-	<1/160
Anti-dsDNA (IU/mL)	-	0.7	-	<30
Anti-SSA (Ro)	-	Positive	-	-
Anti-SSB (La)	-	Positive	-	-
ANA pattern	-	Homogeneous nuclear pattern (AC-1); fine speckled pattern (AC-4)	-	-

**Figure 2:**
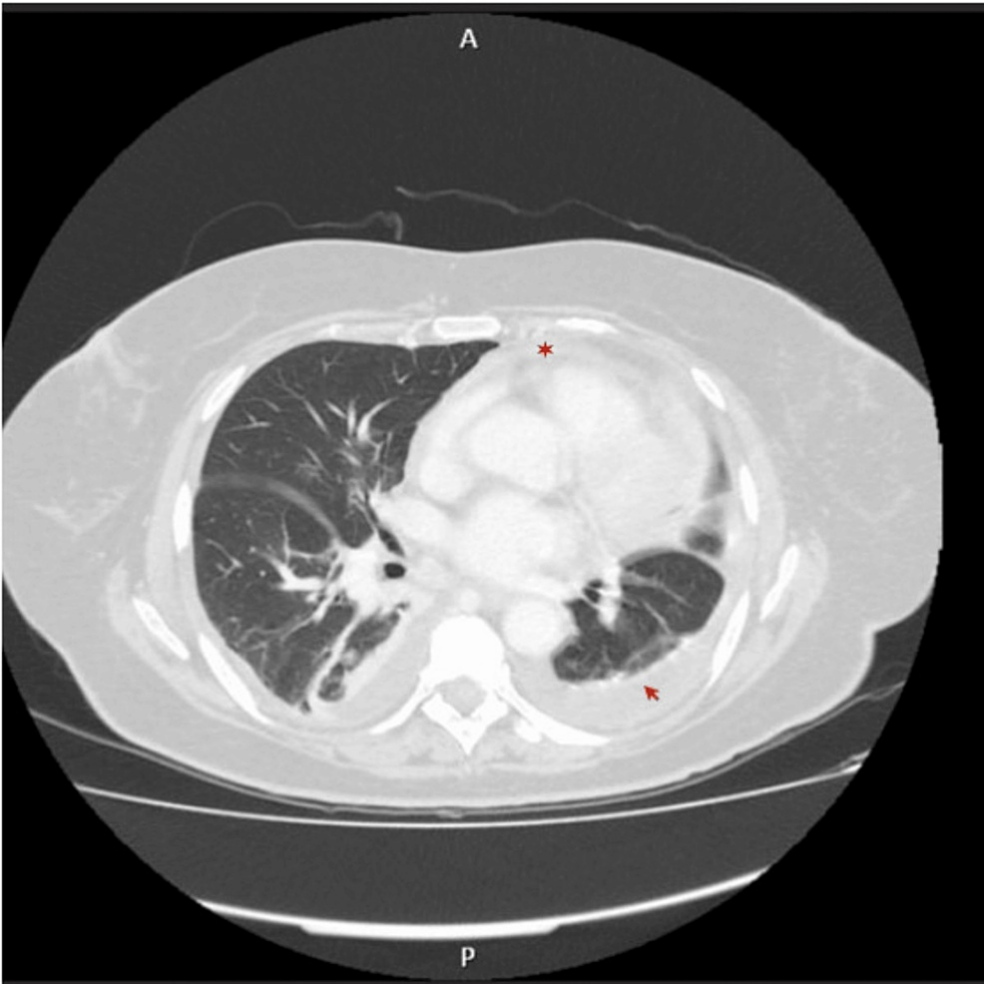
Axial computed tomography (CT) showing mild pericardial effusion (red star) and confirming the pleural effusion (red arrow)

The patient was readmitted for pneumonia with parapneumonic pleural effusion and started empiric antibiotherapy with ceftriaxone and clindamycin, followed by vancomycin and ceftazidime targeted to *Pseudomonas aeruginosa* and methicillin-resistant *Staphylococcus aureus* isolated on sputum. Despite target treatment, the patient remained febrile and oxygen-dependent, with significant adynamia, asthenia, and pleural effusion on chest X-ray. Due to the suspicion of noninfectious inflammatory cause, additional laboratory studies with autoimmunity were requested (Table 1, evolution). Computed tomography (CT) scans of the chest and abdomen were conducted. Immunologic laboratory tests found positive antinuclear antibodies at a titer of 1:640 and positive anti-Sjögren's syndrome-related antigen A (anti-SSA) (Ro), anti-Sjögren's syndrome-related antigen B (anti-SSB) (La), and anti-Ro-52 antibodies. Anti-double-stranded DNA (dsDNA) and rheumatoid factor (RF) were negative. The remaining blood results showed a persistent elevation of acute phase proteins, severe hypophosphatemia, hypokalemia, and hypocalcemia. Chest CT revealed mild pericardial effusion and bilateral pleural effusion with adjacent atelectasis (Figure 2). No other changes were identified such as airway or interstitial lung abnormalities. Left-side thoracentesis was performed, and the biochemical examination of pleural fluid was compatible with exudate, with no neoplastic cells identified. Microbiological study was also negative. Cardiac ultrasound confirmed a thin line of pericardial effusion.

**Figure 3:**
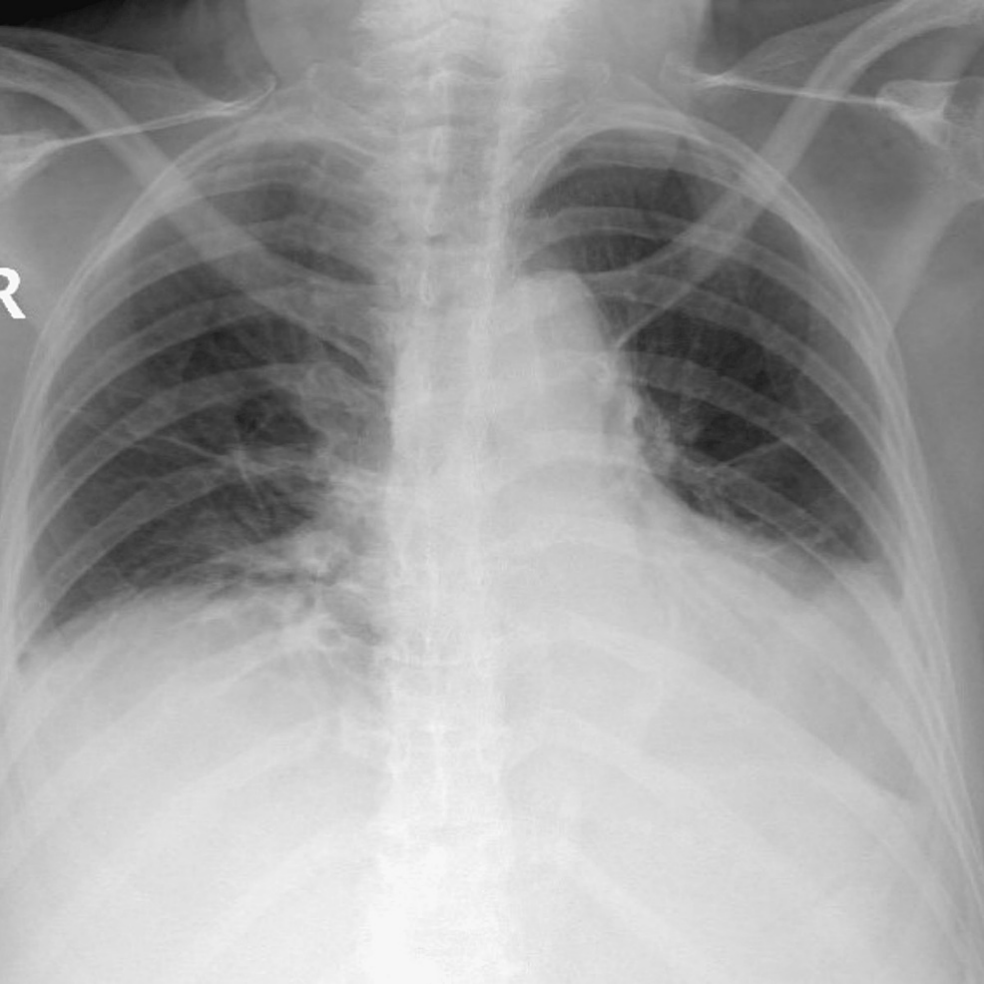
Chest X-ray at discharge Substantial improvement of pleural effusion after corticosteroid onset

These results are highly indicative of pSS with serosal involvement. The patient was started on corticosteroid with methylprednisolone pulses 500 mg/day for three days, followed by an oral taper with prednisolone. The introduction of corticosteroid therapy was associated with fever resolution and the reduction of the pleural effusion (Figure 3). Blood test at discharge is described previously (Table 1, at discharge). Later, the diagnosis of pSS was supported by the Schirmer test. Currently, and after three years of follow-up, the patient does not need immunosuppressive therapy and remains without relevant lung changes in this context.

## Discussion

Sjögren's syndrome (SS) is a chronic autoimmune disorder characterized by the infiltration of lymphocytes into the exocrine glands, primarily affecting the salivary and lacrimal glands. In this clinical case, the latest consensus of the American-European Consensus Group (Table 2) is followed for diagnosing SS. It can occur as primary Sjögren's syndrome (pSS) or in association with other connective tissue diseases, known as secondary Sjögren's syndrome (sSS). Diagnosing SS can be challenging due to the potential overlap with other connective tissue diseases. The manifestations of SS can be categorized as exocrine and non-exocrine, and theoretically, any organ can be affected [4].

**Table 2:** ACR-EULAR 2016 classification criteria for Sjögren's syndrome (SS) A score of ≥4 classifies a patient who meets the following inclusion criteria: ocular and oral dryness (based on AECG questions) or suspicion of SS from the EULAR Sjögren's Syndrome Disease Activity Index (ESSDAI) questionnaire. And it does not have any of the following exclusion criteria: history of head and neck radiation treatment, active hepatitis C infection, AIDS, sarcoidosis, amyloidosis, graft-versus-host disease, and IgG4-related disease ACR-EULAR, American College of Rheumatology-European Alliance of Associations for Rheumatology; AECG, American-European Consensus Group; SSA, Sjögren's syndrome-related antigen A

Item	Item to be scored	Weight
1	Labial salivary gland with focal lymphocytic sialadenitis and focus score of ≥1	3
2	Anti-SSA/Ro-positive	3
3	Ocular staining score of ≥5 (or van Bijsterveld score of ≥4) in at least one eye	1
4	Schirmer test of ≤5 mm/five minutes in at least one eye	1
5	Unstimulated whole saliva flow rate of 0.1 mL/minute	1

Pulmonary involvement is significant in 10%-20% of patients with SS, particularly affecting the airways and pulmonary interstitium [5]. The most common pulmonary manifestation in pSS is interstitial lung disease (ILD), which refers to a group of lung disorders characterized by the inflammation and fibrosis of the lung interstitium. ILD associated with pSS often presents as a nonspecific interstitial pneumonia pattern, leading to progressive dyspnea, cough, and reduced lung function. High-resolution computed tomography (HRCT) plays a crucial role in diagnosing ILD in pSS patients, aiding in treatment decisions and disease monitoring [6].

Lymphocytic interstitial pneumonia (LIP) is another recognized pulmonary manifestation of pSS, characterized by lymphocyte infiltration into the lung interstitium, resulting in interstitial inflammation. LIP can cause respiratory symptoms such as cough, dyspnea, and chest discomfort. HRCT findings typically reveal ground glass opacities and nodules, along with the presence of lymphoid aggregates. In some cases, LIP can progress to more severe forms of ILD [7].

However, serous effusion is a rare manifestation in pSS, with only a limited number of reported clinical cases. Among 349 diagnosed SS patients, 31 (9%) had pulmonary involvement, and five (1%) had pleural effusion, with only two of them having no simultaneous diagnosis of other connective tissue disorders [8]. Another study reported a prevalence of 0% in pSS and 8% in sSS. Pleural fluid in these cases usually exhibits cytochemical, non-pathognomonic, exudative-like features, with lymphocytic cells, normal pH and glucose levels, and decreased levels of adenosine deaminase (ADA) [9,10]. Although robust evidence is lacking, the rational option for treatment is the use of corticosteroids at a dose of 1 mg/kg/day.

As mentioned earlier, the patient experienced a range of chronic symptoms initially attributed to fibromyalgia, which delayed the diagnosis of pSS. We hypothesize that the serosal effusion was triggered by an infectious occurrence and sustained by autoimmunity.

## Conclusions

Primary Sjögren's syndrome is not solely limited to affecting the exocrine glands but also other organs. The recognition and understanding of these extraglandular complications are vital for timely diagnosis, appropriate treatment, and improved patient outcomes. Further research into these manifestations is needed to develop targeted therapies and enhance our ability to manage these complex cases effectively.
